# Vitamin B_12_ as a source of variability in isotope effects for chloroform biotransformation by *Dehalobacter*

**DOI:** 10.1002/mbo3.1433

**Published:** 2024-08-27

**Authors:** Elizabeth Phillips, Katherine Picott, Steffen Kümmel, Olivia Bulka, Elizabeth Edwards, Po‐Hsiang Wang, Matthias Gehre, Ivonne Nijenhuis, Barbara S. Lollar

**Affiliations:** ^1^ Department of Earth Sciences University of Toronto Toronto Ontario Canada; ^2^ Department of Chemical Engineering and Applied Chemistry University of Toronto Toronto Ontario Canada; ^3^ Department of Technical Biogeochemistry Helmholtz Centre for Environmental Research—UFZ Leipzig Germany; ^4^ Present address: Inorganic Chemistry Laboratory University of Oxford Oxford UK; ^5^ Present address: Graduate Institute of Environmental Engineering National Central University Taoyuan City Taiwan

**Keywords:** biotransformation, compound‐specific isotope analysis, dual‐isotope analysis, enzyme kinetics, organohalide respiration, reductive dechlorination

## Abstract

Carbon and chlorine isotope effects for biotransformation of chloroform by different microbes show significant variability. Reductive dehalogenases (RDase) enzymes contain different cobamides, affecting substrate preferences, growth yields, and dechlorination rates and extent. We investigate the role of cobamide type on carbon and chlorine isotopic signals observed during reductive dechlorination of chloroform by the RDase CfrA. Microcosm experiments with two subcultures of a *Dehalobacter*‐containing culture expressing CfrA—one with exogenous cobamide (Vitamin B_12_, B12^+^) and one without (to drive native cobamide production)—resulted in a markedly smaller carbon isotope enrichment factor (*ε*_C, bulk_) for B12^−^ (−22.1 ± 1.9‰) compared to B12^+^ (−26.8 ± 3.2‰). Both cultures exhibited significant chlorine isotope fractionation, and although a lower *ε*_Cl, bulk_ was observed for B12^−^ (−6.17 ± 0.72‰) compared to B12^+^ (−6.86 ± 0.77‰) cultures, these values are not statistically different. Importantly, dual‐isotope plots produced identical slopes of *Λ*_Cl/C_ (*Λ*_Cl/C, B12+_ = 3.41 ± 0.15, *Λ*_Cl/C, B12_− = 3.39 ± 0.15), suggesting the same reaction mechanism is involved in both experiments, independent of the lower cobamide bases. A nonisotopically fractionating masking effect may explain the smaller fractionations observed for the B12^−^ containing culture.

## Graphical abstract

The presence of vitamin B_12_ has a significant effect on carbon isotope effects, consistent with an isotope masking effect. We interpret this in the context of predicted enzyme structures.

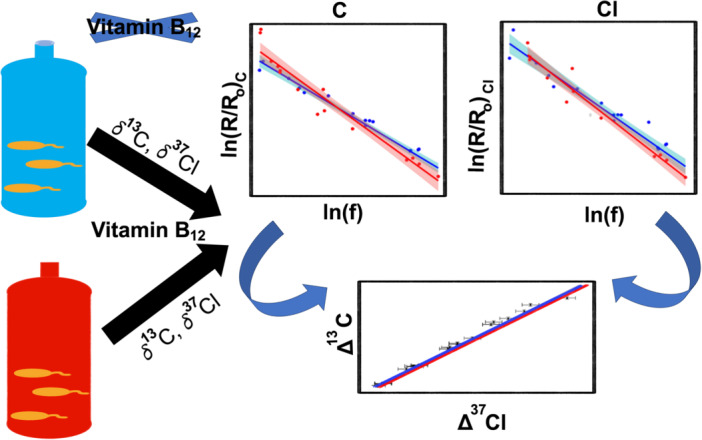

## 1 INTRODUCTION

Chloroform (CF, trichloromethane) has historically been used as an anesthetic and a precursor for refrigerants and fluoropolymers (Holbrook, 2000). CF is a groundwater contaminant found at ∼25% of the United States Environmental Protection Agency priority sites, (USEPA, 2015) a concern due to its carcinogenic effects (IARC, 1999) and toxicity to the central nervous system, liver, and kidneys (Agency for Toxic Substances and Disease Registry, 1997). Contaminant concentrations can decrease via physical processes (e.g., volatilization, dilution, diffusion) and transformation processes, with the latter preferred for site remediation, as transformation removes CF mass and can produce less harmful end‐products (Bulka, Webb, et al., 2023; Justicia‐Leon et al., 2014; Lee et al., 2012; Wang et al., 2022).

Compound‐specific isotope analysis (CSIA) is a powerful tool to identify contaminant transformation in the field and estimate remediation rates and extents based on differences in activation energies between bonds containing exclusively light isotopes of an element (^L^ *E*) and those containing a heavy isotope (^H^ *E*) (Hunkeler et al., 2008). This varying activation energy causes slight differences in reaction rate constants for molecules containing exclusively light (^L^k) isotopes versus one or more heavy (^H^ *k*) isotopes of an element in a reactive position (primary isotope effect) or its vicinity (secondary isotope effect)—that is, the kinetic isotope effect (KIE).

Over the last decade, CSIA has been used to probe transformation mechanisms and kinetics (reviewed elsewhere [Ojeda et al., 2020]; Elsner, 2010). Briefly, the intrinsic KIE magnitude is determined by the transition state structure, which is influenced by the order and manner of bond breakage and formation in the transformation step(s). However, biotransformation processes are comprised of multiple elementary reaction steps, including nontransformation steps such as mass transfer (Bosma et al., 1997) or substrate–enzyme complex formation (Michaelis & Menten, 1913). If a nontransformation step preceding the transformation step is rate‐limiting, the observed KIE may be suppressed, (Jencks, 1969) producing a lower apparent KIE (AKIE), known as a “masking effect.” Identifying masking effects and conditions that influence them are thus important in applying and interpreting CSIA (Ehrl et al., 2018; Nijenhuis et al., 2005; Thullner et al., 2013). Since masking typically affects both elements equally, the ratios of dual‐element isotope effects are insensitive to masking. Hence dual‐isotope plots, where isotopic compositions of two elements within a reactive bond are plotted against each other, can overcome masking effects. These plots generally reflect a linear relationship, with a regression slope (lambda, *Λ*) that yields more direct insight into reaction mechanisms (Ojeda et al., 2020). However, several exceptions (Gafni et al., 2020; Renpenning et al., 2015) have been reported, where *Λ* varies for the same transformation mechanism due to additional rate‐limiting steps that cause isotope fractionation.

Previous investigations of CF biotransformation have shown large and reproducible carbon isotope effects during biodegradation by organohalide‐respiring enrichment culture ACT‐3 CF with ethanol and lactate as electron donors (ACT‐3/EL) (Chan et al., 2012; Heckel et al., 2019; Phillips et al., 2022). A description of ACT‐3 is included in the Supporting Information S1. ACT‐3 CF/EL grown with vitamin B_12_ has similar dual carbon and chlorine isotope effects (*ε*_C_ = −27.91 ± 1.66, *ε*_Cl_ = −4.00 ± 0.20, *Λ* = 6.64 ± 0.14) to abiotic degradation of CF by vitamin B_12_ (*ε*_C_ = −26.04 ± 0.91, *ε*_Cl_ = −4.20 ± 0.26, *Λ* = 6.46 ± 0.20) (Heckel et al., 2019) suggesting a common reaction mechanism. However, a large range of *ε*_C_ (−1.52 to −27.91‰) and *ε*_Cl_ (+2.52 to −6.86‰) have been observed in other CF biotransformation studies, producing different *Λ*_C/Cl_ (6.64 to −1.2) (Chan et al., 2012; Heckel et al., 2019; Lee et al., 2015; Phillips et al., 2022; Soder‐Walz et al., 2022). The cause of variability in observed carbon and chlorine isotope effects for CF biotransformation remains unresolved to date, though isotope effects appear to be enzyme‐specific.

Reductive dehalogenases (RDases) are involved in organohalide respiratory processes and utilize a cobamide cofactor to reduce halogenated substrates (Jugder et al., 2015, 2016; Leys et al., 2013). Cobamides contain a tetrapyrrole ring, a ribose, a centrally chelated cobalt ion, and two axial ligands—an upper and a lower—and are differentiated primarily by the lower ligand/base structure. The three main lower ligand classes are benzimidazoles, phenolics, and purines. Cobalamin (vitamin B_12_) is a cobamide with 5′,6′‐dimethylbenzimidazole as a lower base. ACT‐3 contains *Dehalobacter* sp. CF which possesses the complete cobamide biosynthesis pathway, experimentally verified by Wang et al. (2017), although the native cobamide structure remains unknown and does not correspond to any known cobamides. Different cobamide lower bases in RDases have been shown to affect substrate preferences, growth yields, and dechlorination rates and extent (Keller et al., 2014, 2018; Yan et al. 2012, 2013, 2016), yet how the lower bases exert these controls remains elusive. To date, most studies investigating how different cobamides affect RDases have focused on chloroethene dehalogenating RDases.

This study applies CSIA to investigate the impact of vitamin B_12_ on isotope effects produced during the biotransformation of CF. These results are considered in the context of isotope masking effects, predicted protein–cobamide interactions using structural models, and site remediation significance. Differences in the microbial community in cultures grown with and without vitamin B_12_ were assessed, and a metagenomic analysis was performed to investigate cobamide synthesis pathways in the B_12_−free culture. These findings inform how environmental conditions such as cobamide limitation should be considered when using CSIA to evaluate in situ degradation.

## 2 EXPERIMENTAL PROCEDURES

### 2.1 Cultures and growth conditions

Details about the parent culture (ACT‐3) are provided in Supporting Information S1. B12^+^ experiments are from Phillips et al. (2022) where cultures and experimental setup are described. ACT‐3 contains strain CF that dechlorinates CF to dichloromethane (DCM) linked to growth using CfrA (Grostern & Edwards, 2006). A subculture of ACT‐3, CF sub (B_12_^−^), was maintained on CF for 4 years in mineral medium (modified from Edwards and Grbić‐Galić [1994] without vitamin B_12_). Two subcultures were scaled up to 2 L with simultaneously increasing CF concentrations to 1 mM: one from ACT‐3 CF/EL parent culture in medium containing cobalamin (B_12_^+^) (Phillips et al., 2022) and the second from CF sub B_12_^−^, maintained medium without the addition of vitamin B_12_ (B_12_^−^). Cell density was not measured, but cultures were visibly cloudy and consistently degrading 1 mM of CF within 1 week.

### 2.2 Analytical methods

Detailed analytical methods and calculations are presented in Supporting Information S1. Briefly, CF and DCM concentrations were quantified using a Varian 3400 gas chromatograph (GC) with a flame ionization detector (FID). Three‐point calibration curves (concentrations 1.2, 0.6, 0.12 mM, *n* = 3 for each standard) for CF and DCM were prepared and checked daily. Uncertainty was quantified using daily standard reproducibility (*n* = 9), which was within typical GC/FID uncertainty, ±5%. *δ* ^13^C was measured on a Finnigan MAT 252 isotope–ratio mass spectrometer interfaced with a Hewlett‐Packard 6890 GC and combustion oven. Isotopically characterized laboratory working standards of CF (*δ* ^13^C = −49.8 ± 0.1‰) and DCM (*δ* ^13^C = −39.6 ± 0.3‰) were injected daily to ensure accuracy. The total *δ* ^13^C uncertainty is ±0.5‰ incorporating both accuracy and reproducibility (Sherwood Lollar et al., 2007). *δ* ^37^Cl was measured on a Neptune MC‐ICPMS (Thermo Fisher Scientific) interfaced with a Thermo Scientific Trace 1310 GC after the method of Renpenning et al. (2018) Three offline‐characterized in‐house standards were used to normalize sample measurements on the standard mean ocean chloride (SMOC) scale and ensure accuracy. The maximum reproducibility (1σ) observed for sample and control measurements was within ±0.3‰.

CSIA measures stable isotope ratios of an element (*R* = ^H^ *E*/^L^ *E*), typically expressed in *δ* notation (Equation 1):

(1) $${\text{δ}}^{H}E(\text{‰})={(R}_{\text{sample}}/R_{\text{standard}})-1,$$

where *R*_standard_ is the isotopic ratio of an international reference standard for the element of interest (V‐PDB for carbon, SMOC for chlorine [Hunkeler et al., 2008]). Isotopic enrichment factors were calculated using the Rayleigh model (Equation 2):

(2) $$1000\times \ln(R/R_{o})=\varepsilon_{E}\times \ln(f),$$

where in contaminant hydrogeology *R*_o_ is the initial isotopic composition of the parent contaminant, *f* is the fraction of the original contaminant remaining, and *ε*_E_ is an isotopic enrichment factor, the magnitude of isotopic fractionation for a given reaction.

### 2.3 Community composition

To determine the microbial community composition of ACT‐3 CF/B12^+^ and ACT‐ CF/B12^−^, DNA samples were taken from each culture and extracted using the Kingfisher Duo Prime MagMax microbiome kit (Thermo Fisher Scientific). Amplicon sequencing of the 16S ribosomal RNA gene V6–V8 region was performed by Genome Quebec as previously described and run through a previously established QIIME2 pipeline (Bolyen et al., 2019; Bulka, Webb, et al., 2023).

### 2.4 Metagenomic sequencing, assembly, and cobalamin synthesis search

The ACT‐3 CF culture metagenome was sequenced and assembled as previously described, (Bulka, Picott, et al., 2023) and a detailed description is pending. Briefly, 300 mL of the ACT‐3 CF culture was sampled and pelleted twice in November 2020 for DNA extraction as described above. DNA was sequenced by Genome Quebec using both Illumina MiSeq and PacBio Sequel II technologies. Illumina reads were trimmed using Trimmomatic and FastQC (Andrews et al., 2023; Bolger et al., 2014). Long and short reads were assembled using hybridSPAdes (Antipov et al., 2016). Additional quality control, mapping and binning were performed using Anvi'o Snakemake (Eren et al., 2015, 2021; Shaiber et al., 2020). The ACT‐3 CF metagenome was deposited publicly under BioProject PRJNA80805, at accession JAVKYP000000000. Contigs were annotated using MetaErg, (Dong & Strous, 2019) and annotations were searched for hidden Markov models representing anaerobic cobalamin biosynthesis genes (Supporting Information S1: Table S3) (Lu et al., 2020).

### 2.5 Protein models

Protein models were produced for CfrA from *Dehalobacter* sp. CF (protein accession: AFV05253, UniProt ID: K4LFB7) (Tang et al., 2016). CfrA models were obtained using the AlphaFill online server, (Hekkelman et al., 2023) which takes AlphaFold (Jumper et al., 2021) models from a UniProt database and transplants ligands into the model based on homologous crystal structures. CfrA models with two [4Fe‐4S] clusters and either cobalamin or *norpseudo*‐B_12_ transplanted were downloaded from AlphaFill (Hekkelman et al., 2023). The YASARA (Krieger et al., 2009) energy minimization server was used for model optimization and water addition. The TAT signal peptide sequence was removed from the structure by predicting the cleavage sites using SignalP 6.0, (Teufel et al., 2022) and trimming the sequence to match the cut‐site suggested for the homologous enzyme, TmrA (Jugder et al., 2017). The PceA model is visualized as the monomer of structure 4UR0 in the Protein Data Bank (Bommer et al., 2014). Solvent accessibility was assessed using access channels predicted using the CAVER 3.0 plugin in PyMOL v2.3.4, only major channels accessing the cobamide binding site were kept. Polar contacts were assessed, and images were produced using PyMOL v2.3.4.

## 3 RESULTS

### 3.1 Carbon and chlorine isotope effects in B12^+^ and B12^−^

**Figure 1:**
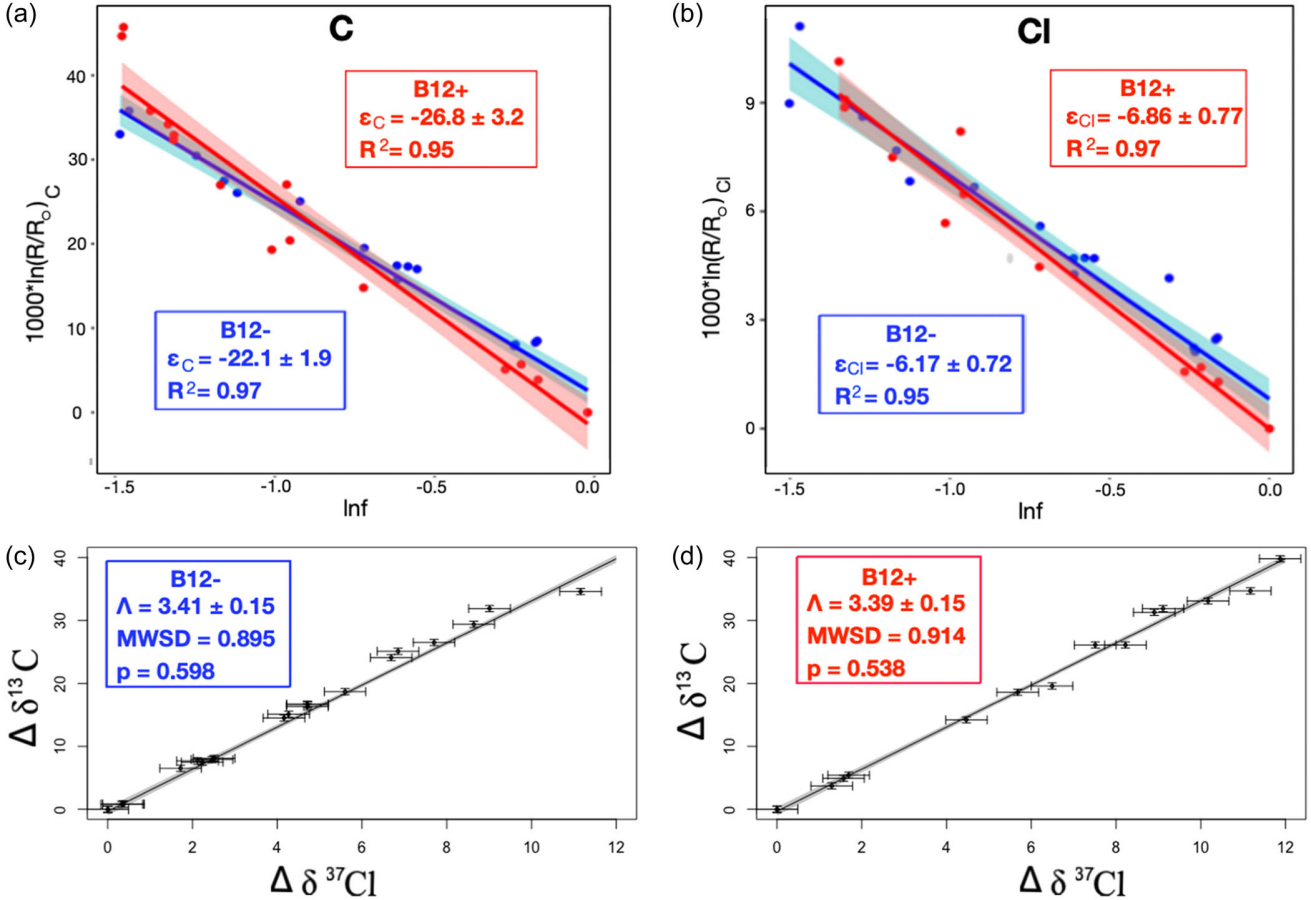
Rayleigh plots (a, b) for the B12− (blue) and B12+ (red) subcultures (from Phillips et al. [2022]) for carbon (a) and chlorine (b) and dual‐isotope plots (c, d) for B12− (c) and B12+ (d). Shading represents 95% confidence interval of the slope (see text for details). The mean square of weighted deviations and the associated *p*‐value are shown for dual‐isotope plots, regressed using York regression, to assess model fit (detailed discussion provided in Ojeda et al. [2021]).

No significant change in *δ* ^13^C (Supporting Information S1: Figure S1), *δ* ^37^Cl (Supporting Information S1: Figure S2), or mass (Supporting Information S1: Figures S3 and S4) was observed for sterile controls. CF was transformed at similar rates within experimental replicates; to *f* = ∼0.2 within 6 days for B12^+^ (Supporting Information S1: Figure S3), and to ∼0.15 within 7 days for B12^−^ (Supporting Information S1: Figure S4). Biotransformation of CF produced significant carbon and chlorine isotope fractionation in both B12^+^and B12^−^, with enrichment in ^13^C (Δ*δ* ^13^C) of up to 45.1‰ and 34.6‰ and Δ*δ* ^37^Cl of up to 13.4‰ and 11.2‰ in B12^+^ and B12^−^, respectively. Equation (2) was used to calculate *ε*_C, bulk_ and *ε*_Cl, bulk_ from the data in Figure 1a,b. Data from reaction progress *f* < 0.2 were not included in *ε*_bulk_ calculations as higher uncertainty in these data can significantly impact calculated *ε*_bulk_ (Bigeleisen & Allen, 1951; Mundle et al., 2013). Correlation coefficients (*R* ^2^ values) were ≥0.95, consistent with observations for biotransformation‐associated isotope experiments in the literature. Based on recent recommendations, (Ojeda et al., 2019, 2021) the York method (York, 1966, 1969) was used for linear regression of the data in Figure 1c,d to calculate *Λ*_C/Cl_. The associated mean square of weighted deviates and *p*‐values (Figure 1c,d) indicate the model is appropriate for the data. Statistical tests (*z* tests) were used to compare regression slopes (Ojeda et al., 2019). Figure 1 shows Rayleigh and dual‐isotope plots for B12^+^ and B12^−^. B12^−^ produced a statistically different *ε*_C_ (−22.1 ± 1.9‰) versus B12^+^ (*ε*_C_ = −26.8 ± 3.2‰), with *p* < 0.05 (Table 1). The difference in *ε*_C_ between B12^+^ and B12^−^ is particularly significant considering the consistency of the value for B12^+^ *ε*_C_ (−26.8 ± 3.2‰) with earlier studies of CF biotransformation by ACT‐3 (Chan et al., 2012; Heckel et al., 2019) (Supporting Information S1: Table S1). Further, the corresponding B12^+^ AKIE_C_ (1.0275 ± 0.0034) is consistent with the theoretical KIE_C_ for C–Cl bond cleavage, 1.03, calculated using theoretical semiclassical Streitwieser Limits assuming 50% bond cleavage in the transition state (Huskey, 1991). Both *ε*_C_ and *ε*_Cl_ are lower for B12^−^, although *ε*_Cl_ values are not statistically different based on hypothesis tests (Table 1). However, a lower *p*‐value (0.158) is produced for statistical tests of *ε*_Cl_, particularly when compared to the *p*‐value for *Λ*_C/Cl_ (0.872; Table 1), indicating a lower probability that the *ε*_Cl_ values represent the same underlying “true” value. The absence of exogenous vitamin B_12_ in B12^−^ may indeed cause *ε*_Cl_ suppression that is unresolvable relative to the *δ* ^37^Cl uncertainty, that is, the true underlying *ε*_Cl_ values are different, yet high uncertainty in the regression slopes (95% confidence interval) does not allow us to statistically differentiate them at the designated confidence level (*α* = 0.05). Importantly, differences in *ε*_C_ and *ε*_Cl_ do not result in statistically different *Λ*_C/Cl_ values between B12^+^ and B12^−^ (Figure 1c,d and Table 1).

**Table 1:** Comparison of *ε*_C_ and *ε*_Cl_, calculated AKIE values and *Λ*_C/Cl_ values between the B12^+^ and B12^−^ subcultures.

	B12− Expt	B12+ Expt	p Values	Statistically different (*α* = 0.05)
*ε*_C_	−22.1 ± 1.9‰	−26.8 ± 3.2‰	8.21E−03	Yes
AKIE_C_	1.0226 ± 0.0020	1.0275 ± 0.0034		
*ε*_Cl_	−6.17 ± 0.72‰	−6.86 ± 0.77‰	0.158	No
AKIE_Cl_	1.0189 ± 0.0022	1.0210 ± 0.0011		
*Λ*_C/Cl_ (York)	3.41 ± 0.15	3.39 ± 0.15	0.872	No

*Note*: Error for AKIE is propagated through Equation (3) using standard error propagation. All uncertainty is reported to two significant figures and the measured or calculated value is reported to the same digit as the uncertainty. Statistically different regression slopes (determined by p‐values from Z tests) are indicated by green shading while regression slopes that are not statistically different are indicated by orange shading)

Abbreviation: AKIE, apparent kinetic isotope effect.

### 3.2 Community composition in B12^−^ versus B12^+^

*Dehalobacter* is the most abundant bacterial genus in ACT‐3 CF, comprising ∼75% of bacterial reads in both subcultures (Supporting Information S1: Figure S5). All ASVs are summarized in Supporting Information S1: Table S4. Notable differences between the two culture conditions include an increased abundance of an *Acinetobacter* sp. and a *Desulfovibrio* sp. in the B_12_−free condition, as well as an archaeal shift from 100% *Methanosphaerula* to 63% *Methanosaeta* (Supporting Information S1: Figure S5). Interestingly, *Methanosaeta* are acetoclastic methanogens, while *Methanosphaerula* is strictly hydrogenotrophic, suggesting an increased production of acetate by the bacterial species in the B_12_−free culture (Cadillo‐Quiroz et al., 2009; Patel & Sprott, 1990). *Desulfovibrio* is known to produce acetate and exist in syntrophic relationships with methanogens (Stolyar et al., 2007). *Acinetobacter* also uses acetate as a carbon source and may be consuming this byproduct from *Desulfovibrio* (Pirog & Kuz'minskaya, 2003).

Additionally, both *Acinetobacter* and *Desulfovibrio* have been previously shown to produce cobamides. *Desulfovibrio vulgaris*, for example, has been shown to possess all necessary genes for cobalamin synthesis (Hildenborough et al., 2008). Additionally, some *Acinetobacter* strains encode the necessary genes for nucleotide loop assembly and can convert cobinamide precursors into different cobamides, including cobalamin (Villa & Escalante‐Semerena, 2022). A closer metagenomic look at ACT‐3 CF was performed to provide more insight into the role of these microorganisms in B12^−^.

### 3.3 Cobamide pathways identified via metagenomic sequencing

Partial cobamide biosynthesis pathways were found in 102 genera and 59 families in the ACT‐3 CF culture. All hits and their predicted taxonomies are shown in the attached file (Supporting Information S1: Table S3). Taxa with more than 10 different biosynthetic genes, including *Dehalobacter* and *Desulfovibrio*, are summarized in Table 2. Other potential cobalamin producers in ACT‐3 CF are *Sporomusa* and UBA5314 of the Syntrophomonadaceae family. *Acinetobacter* was not detected in the metagenome, and metagenome sequencing of B12^−^ should be performed for a more thorough analysis of this subculture.

**Table 2:** Summary of key genera encoding the cobamide biosynthetic pathway in the ACT‐3 CF metagenome.

Step code	HMM	g_*Sporomusa*_C	g_*Dehalobacter*	g_*Desulfovibrio*_F	f__Syntrophomonadaceae; g_UBA5314	c_Peptococcia; g_UBA5318	f__Syntrophomonadaceae; g_UBA4844	f__Anaerovoracaceae; g_UBA7709	g_*Methanosphaerula*	g_*Propionicimonas*	g_*Sedimentibacter*
CbiD	PF01888	1	1	1	2	3	1	4	1		3
CbiG	PF01890	1	1	1	1	3		1	1		
CbiK/X	PF01903	2	1	1	2		1	2	1	4	1
CbiC	PF02570	1	1	1	1	2			1	1	1
CbiJ	PF02571	1	1		1	1	1	3			2
CbiK/X	PF06180	5		2	2		1	4			1
CbiD	TIGR00312	1	1	1	1	2	1	1			
CbiA	TIGR00379	1	1	1	1	2	1		2	1	
CbiJ	TIGR00715	1	1		1	1	1	1			
CbiF	TIGR01465	1	1	1	1	1	1				
CbiH	TIGR01466	1	1	1	1	2	1	1	1		1
CbiL	TIGR01467	1	1	1	2	2		2			
CobA	TIGR01469	1	1	1					1	1	
CbiE	TIGR02467	1	1	1		2	1	1		1	1
CbiT	TIGR02469	1	1	1	1	3	1	1		1	
CobA	PF01923	1						1		2	1
CobU	PF02283	1	1	1	2			1		1	
CobS	PF02654	2	1	1	1			2	1	1	
CobD	PF03186	1	1	1	1	1	1	1	1		1
CobQ	TIGR00313	1	1	1	1	1		2	1	1	
CobS	TIGR00317	1	1		1				1		
AcbPsyn	TIGR00380	1	1		1	1	1		1		1
CobA	TIGR00636	1								1	
CobA	TIGR00708	1						1		1	
ThrP_dc	TIGR01140	1	1	1							
CobT	TIGR03160	1	1	1	1			1		1	
CobC/Z	TIGR03161										
CobC/Z	TIGR03162				1						

*Note*: Gene copies are highlighted in green, and optional steps are marked with gray. Genera derived from uncultivated bacterial and archaeal (UBA) taxa are also labeled with their best‐named rank. The steps are described in Supporting Information S1: Table S2. For all hits, see Supporting Information S1: Table S3.

Abbreviations: CF, chloroform; HMM, hidden Markov models.

## 4 DISCUSSION

### 4.1 Isotope masking effects in B12^−^

*ε*_C_ Values are statistically different for CF biotransformation by B12^+^ versus B12^−^ (Table 1). While hypothesis tests do not support statistically different *ε*_Cl_ values at a confidence level of 0.05, this result is partly due to the uncertainty in *ε*_Cl_ which is >∼±0.7‰ (Table 1). The absolute differences between *ε*_C_ (4.7‰) and *ε*_Cl_ (0.69‰) for B12^+^ and B12^−^ are considerably larger than differences observed for chlorinated ethene biotransformation with different cobamide types, where Δ*ε* were <0.3‰ for both carbon and chlorine (see discussion below). Overall, the carbon and chlorine isotope results suggest possible masking of isotope effects in B12^−^. The available data however is insufficient to identify the rate‐limiting step causing masking. Both subcultures were derived from the same parent culture (ACT‐3 CF) maintained under whole‐cell conditions. Thus, common rate‐limiting steps that may lead to masking, such as diffusion through the cell membrane, (Nijenhuis et al., 2005; Renpenning et al., 2015) are unlikely to cause masking in B12^−^ relative to B12^+^. Although evolutionary changes in the cell membrane between the two cultures cannot be ruled out, this is unlikely since there was no evolutionary pressure on the organisms to evolve different mechanisms for diffusion through the cell membrane. Commitment to catalysis (Cleland et al., 1976; Northrop, 1981), where enzyme–substrate binding is the rate‐limiting step, is a possible cause of masking in B12^−^. In such cases, masking is proportional to the ratio of the rate constant for transformation (*k*_2_) and the enzyme–substrate dissociation step (*k*_−1_), also known as the commitment factor (*C*) (Cleland et al., 1976; Northrop, 1981) (Equation 3).

(3) $$\text{AKIE}\;=\frac{\text{KIE}+C}{1+C},\text{where}\;C=\text{commitment factor}=k_{2}/k_{-1}.$$

Because the difference in growth conditions between the two subcultures is the presence of vitamin B_12_, a masking effect caused by a difference in *k*_2_/*k*_−1_ is a reasonable mechanism to explain the observations. The difference in *k*_2_/*k*_−1_ may arise from altered enzyme–substrate dissociation (i.e., changes in *k*_−1_) due to differences in the protein structure with different cobamides (discussed in more detail in the context of the CfrA structural prediction below). Other mechanisms that could explain the results include considerations of crystallization structures. Those available for RDases (Bommer et al., 2014; Payne et al., 2015) show the cobamide in base‐off coordination (i.e., the lower base and the centrally chelated cobalt are not coordinated). Crystal structures do not account for enzyme dynamics, and the lower base could play a role in conformational changes during catalysis, in which case, dissociation of the lower ligand from the cobalt ion, causing a masking effect, cannot be ruled out. Any one of these three scenarios could control the reaction kinetics of the enzyme–substrate binding step, and any would be consistent with a commitment to catalysis acting as the underlying control on the differences in observed isotope effects between these two cultures.

Consistent *Λ*_C/Cl_ values suggest a common underlying reaction mechanism in both subcultures and that any masking in B12^−^ compared to B12^+^ (Figure 1a,b) is due to a nonfractionating additional rate‐limiting step. Based on dual carbon/chlorine isotope results from Heckel et al. (2019) this reaction likely proceeds via bimolecular nucleophilic substitution (S_N_2). *Λ*_Cl/C_ values reported here (3.41 ± 0.15 for B12^−^ and 3.39 ± 0.16 for B12^+^) and in previous studies for reductive dechlorination of CF (*Λ*_Cl/C_ ∼ 6–8, for a discussion on this variation see Supporting Information S1: Section 3) are significantly different from other engineered remediation strategies such as persulfate oxidation (17 ± 2) and alkaline hydrolysis (13.0 ± 0.8) (Ojeda et al., 2020). Thus, dual‐isotope analysis can reliably differentiate CF reductive dechlorination from other transformation pathways, regardless of whether vitamin B_12_ is present or not.

Despite the likely presence of masking affecting the observed carbon isotope effects for B12^−^ relative to B12^+^ and the importance of this finding with respect to interpreting the details of reaction mechanisms, the effect of these different *ε*_C_ values on the calculation of the extent of degradation is minor. For example, using both values, we can estimate the fraction of contaminant remaining (using Equation [3] with propagated error using standard error propagation and a *δ* ^13^ *C*_o_ = −49.9‰ (*δ* ^13^ *C*_o_ in the B12^−^ experiment)). At the first time step where *δ* ^13^ *C* = −49.1‰, the corresponding calculated *f* (in %) using *ε*_C_ = −22.1 ± 1.9‰ (B12^−^) is between 93% and 100%, and using *ε*_C_ = −26.8 ± 3.2‰ (B12^+^) the calculated range is 94%–100%. At the other end of the spectrum, a similar calculation can be applied for the most enriched values observed in the B12^+^ experiment, specifically *δ* ^13^ *C* = −3.7‰. In this case, the calculated estimates for B12^−^ and B12^+^ range from 9% to 14% and 13%–21%, respectively. Within uncertainty (based on the calculation error propagated through Equation [3] using standard error propagation), these ranges overlap, indicating that either *ε*_C_ value can provide a reasonable basis to calculate the extent of biodegradation and from that, to derive CSIA‐based biodegradation rates consistent with the best practice EPA guidelines (Jugder et al., 2015).

### 4.2 Effects of cobamide

**Figure 2:**
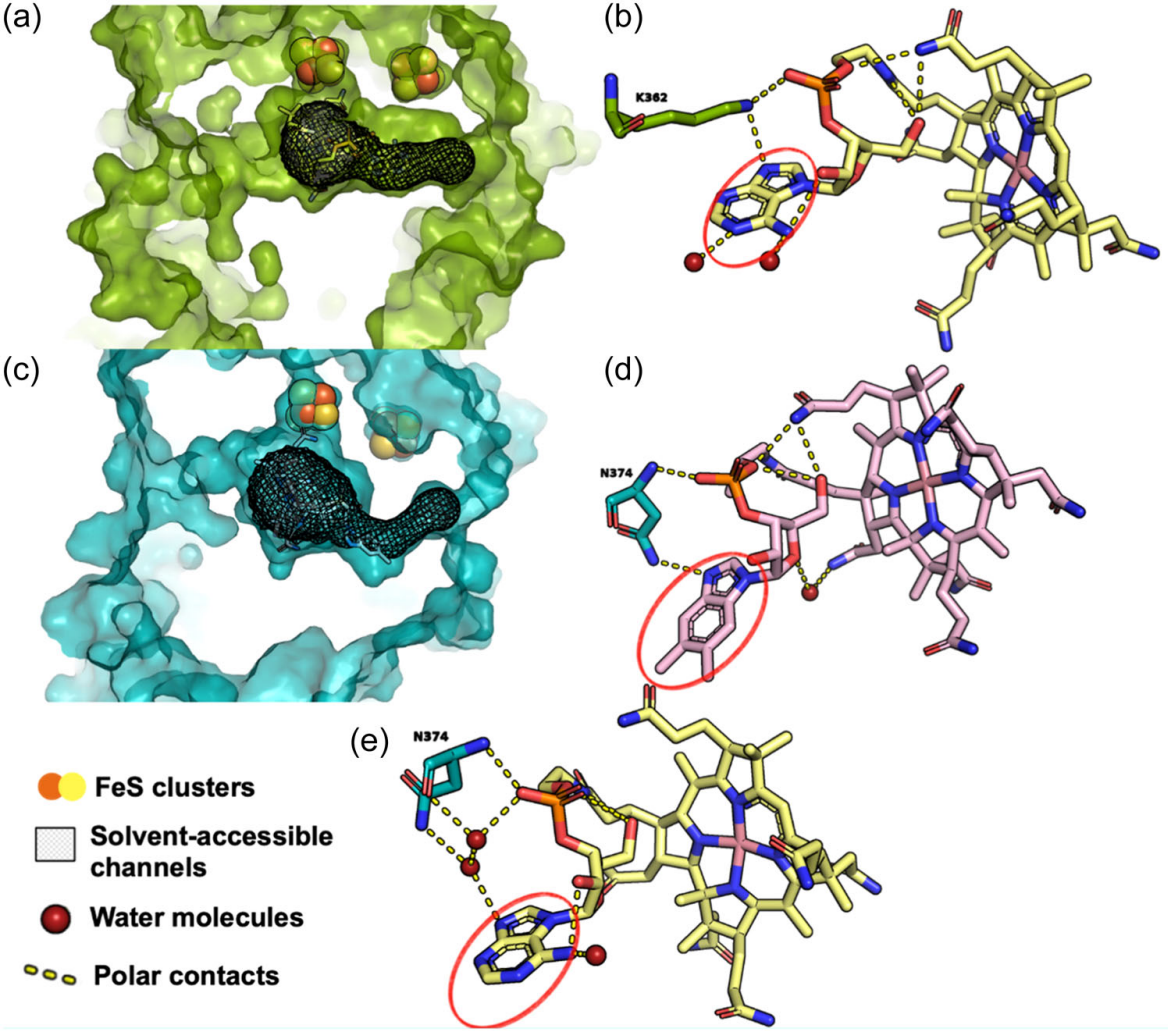
Comparison of protein structure of PceA from *Sulfurospirillum multivorans* (Bommer et al., 2014) (a, b) and AlphaFold model of CfrA obtained from Alphafill with cobamide cofactors (c–e). Predicted solvent‐accessible channels are shown using mesh shading in a profile view of the interior protein surface. Red circles show the location of the cobamide lower base. (a) PceA structure with native cobamide (*norpseudo*‐B12) showing solvent‐accessible channels to the active site. (b) PceA structure with native cobamide showing water molecules (red spheres) and polar contacts (yellow dashed lines). (c) AlphaFold predicted CfrA structure and water accessible channels. (d) AlphaFold predicted CfrA structure with vitamin B_12_ (pink structure) as a cobamide, docked using AlphaFill, including predicted water molecules and polar contacts. (e) AlphaFold predicted CfrA structure with *norpseudo*‐B_12_ (yellow structure) as a cobamide, docked using AlphaFill, including predicted water molecules and polar contacts.

The structure and microbial origin of the cobamide in CfrA in B12^−^ is unknown as the native *Dehalobacter* strain CF cobamide has eluded characterization (Wang et al., 2017) and due to many cobamide biosynthesis genes across multiple taxonomies encoded by the ACT‐3 CF metagenome (Table 2). It may be produced by *Dehalobacter* strain CF itself or scavenged from other organisms in the culture, such as *Desulfovibrio* or *Acinetobacter* (Supporting Information S1: Figure S5). However, the different *ε*_C_ values observed suggest that differences in enzyme activity occur related to the presence/absence of exogenous vitamin B_12_. A previous study using PceA grown in *Sulfurospirillum multivorans* with two different norcobamides (“nor” indicates a lack of a methyl linking moiety compared to cobamides), *nor*‐B12 versus *S. multivorans* native cobamide, *norpseudo*‐B12, revealed no differences in *ε*_C_, *ε*_Cl_, or *Λ* with different norcobamides in PceA (Renpenning et al., 2014). A separate study (Buchner et al., 2022) also showed no variability in carbon, chlorine, or dual‐isotope effects in PceA with different supplied cobalt species. Keller et al. (2018) used nuclear magnetic resonance (NMR) spectroscopy for a structural analysis of norcobamides with crystal structures of PceA, showing water accessibility of the cobamide lower base. These authors proposed substitutions of the lower base are accommodated by changes in water structure rather than changes in the base position or reorientation of the surrounding side chains (Keller et al., 2018). The PceA crystal structure (Bommer et al., 2014) (Figure 2a) shows water accessibility of the lower base, interpreted by overlapping solvent‐accessible channels (mesh shading in Figure 2a) with the lower base of the cobamide, in agreement with Keller et al. (2018) Figure 2b shows interactions between the lower base (circled in red) with water molecules (red spheres).

Importantly, in this study, the CfrA structural prediction (Figure 2c) shows more restricted solvent‐accessible channels compared to the PceA structure. More highly confined channels within the CfrA binding pocket could restrict solvent access to the lower base in CfrA, suggesting changes in the lower base of CfrA are accommodated by a change in protein structure rather than water structure. Using the AlphaFill (Hekkelman et al., 2023) and YASARA (Krieger et al., 2009) energy minimization servers, cofactor and water molecule locations and interactions can be combined with predicted protein structures from AlphaFold (Jumper et al., 2021). Figure 2d shows the predicted CfrA structure with vitamin B_12_. Although a water molecule is present, there are no predicted polar contacts between the water molecule and the lower base of vitamin B_12_, in agreement with this hypothesis. When CfrA is docked with *norpseudo*‐B12 using AlphaFill (Figure 2e), polar contacts are predicted between the cobamide and with water and protein residues. Though the native cobamide in CfrA is unknown, this docking prediction suggests a varying mechanism to accommodate different cobamides in CfrA, involving protein residues or water. A change in polar contacts involving protein residues could result in changes in protein structure with different cobamides. Changes in protein structure could plausibly alter substrate binding to enzymes when different cobamides are incorporated (e.g., here—more efficient binding in CfrA with vitamin B_12_ compared to native cobamide). If structural changes indeed result in more efficient binding with vitamin B_12_ versus the unknown cobamide, this would explain the lower isotope effects observed for B12^−^ versus B12^+^. These findings suggest a basis for the different findings from Renpenning et al.'s (2014) for tetrachloroethene (PCE), compared to those observed here for CF. Additional experimental validation of this hypothesis and predicted CfrA model through determining a CfrA crystal structure will be important for future work.

These results demonstrate that the underlying causes controlling variation in AKIEs should be investigated when studying different cultures in laboratory experiments. Using the Rayleigh equation to identify and evaluate degradation rates and extents can be improved with accurate estimates of *ε*_E_ and *Λ*. Most importantly, the results here demonstrate that a detailed understanding of controls on *ε*_E_ and *Λ* in transformation reactions, even at the level of lower base substitutions, can be gleaned from integrating CSIA in culture‐based experiments with protein structural models. Further, this work reinforces the power of a dual‐isotope approach for evaluating masking effects and providing a basis for robust identification of transformation pathways even if masking effects occur. Along with other recent studies on the effects of active site residues, (Gafni et al., 2020) this study highlights the importance of detailed integration of the structures and activity of proteins with information on reaction efficiency provided by naturally occurring compound‐specific isotope effects as a dual‐pronged approach to understanding the details of contaminant transformation relevant to both experimental and field‐based studies.

## AUTHOR CONTRIBUTIONS

**Elizabeth Phillips**: Conceptualization; investigation; writing—review and editing. **Katherine Picott**: Conceptualization; visualization; investigation; writing—review and editing. **Steffen Kümmel**: Investigation; writing—review and editing. **Olivia Bulka**: Writing—review and editing; investigation. **Elizabeth Edwards**: Conceptualization; writing—review and editing; supervision. **Po‐Hsiang Wang**: Investigation; writing—review and editing. **Matthias Gehre**: Investigation. **Ivonne Nijenhuis**: Investigation; writing—review and editing. **Barbara Sherwood Lollar**: Investigation; conceptualization; supervision; writing—review and editing.

## CONFLICT OF INTEREST STATEMENT

None declared.

## ETHICS STATEMENT

None required.

## Supporting information

Supporting information.

Supporting information.

Supporting information.

Supporting information.

Supporting information.

## Data Availability

The data that support the findings of this study are openly available in the NCBI repository at https://www.ncbi.nlm.nih.gov/bioproject/PRJNA80805.
