# Supplementary material for: Vitamin B12 as a source of variability in isotope effects for chloroform biotransformation by Dehalobacter
Source: Microbiologyopen. 2024 Aug 27;13(4):e1433. doi: 10.1002/mbo3.1433 (PMC11348799; doi:10.1002/mbo3.1433)
Supplement: Supplementary file 4 — Supporting information. [file MBO3-13-e1433-s002.docx]

**Supporting Information**

**Vitamin B_12_ as a Source of Variability in Isotope Effects for Chloroform Biotransformation by *Dehalobacter***

**Running Title:** Vitamin B_12_ Impacts Carbon and Chlorine Isotope Effects

*Elizabeth Phillips*^†||^*, Katherine Picott*^§^*, Steffen Kümmel*^‡^*, Olivia Bulka*^§^*, Elizabeth Edwards*^§^*, Po-Hsiang Wang*^§^*^,#^, Matthias Gehre*^‡^*, Ivonne Nijenhuis*^‡^*, Barbara Sherwood Lollar*^†^

^†^ Department of Earth Sciences, University of Toronto, 22 Ursula Franklin Street, Toronto, Ontario M5S 3B1, Canada

^§^Department of Chemical Engineering and Applied Chemistry, University of Toronto, 200 College Street, Toronto, Ontario M5S 3E5, Canada

^‡^Department of Technical Biogeochemistry, Helmholtz Centre for Environmental Research – UFZ, Permoserstrasse 15, 04318 Leipzig, Germany

^||^Current address: Inorganic Chemistry Laboratory, University of Oxford, Oxford, United Kingdom
#Current address: Graduate Institute of Environmental Engineering, National Central University, Taoyuan City, Taiwan

Containing 17 pages including 5 figures (Figures S1-S5), 2 tables (Tables S1-S2, see S-3 and S-4 in attached file), detailed culture information and microcosm setup, detailed analytical methods, and a discussion of different chlorine isotope results between studies

* Corresponding author: barbara.sherwoodlollar@utoronto.ca

## Culture Information and Microcosm Setup

The parent culture (ACT-3) has been maintained for more than 10 years in minimal mineral salt medium amended with 1,1,1-TCA as an electron acceptor, with an electron donor mix of either MEAL (methanol, ethanol, acetate, and lactate) or EL (ethanol, lactate) as previously established^1^. ACT-3 CF/EL is a microbial culture originally enriched from a site contaminated with 1,1,1-trichloroethane (1,1,1-TCA), that has been maintained in the laboratory, with CF as the electron acceptor, and ethanol and lactate as the electron donors as previously described^2,3^.

ACT-3 CF/EL contains *Dehalobacter* sp. CF, which contains the RDase CfrA, capable of dechlorinating CF, 1,1,1-TCA, and 1,1,2-TCA.^3^ CfrA dechlorinates CF to dichloromethane (DCM) and 1,1,1-TCA to 1,1-dichloroethane (1,1-DCA) via hydrogenolysis.^3^ *Dehalobacter* sp. CF contains the complete cobinamide biosynthesis pathway, experimentally verified by Wang et al.^4^, although the specific corrinoid involved remains unknown. In strain CF, when cyanocobalamin (vitamin B_12_) is provided, vitamin B_12_ is preferentially incorporated into CfrA rather than native cobamide synthesis.^4^ Furthermore, if DMB (the lower ligand of B_12_) is provided, vitamin B_12_ is preferentially synthesized and incorporated into CfrA with similar activity to vitamin B_12_ amended cultures^4^, while strain CF without supplementation of cobalamin or DMB shows significantly lower activity.^4^ Despite efforts to characterize the native cobamide of strain CF, the lower base is unknown, but it is thought to have a purine structure.^4^

### Microcosm Setup.

For B12^+^ and B12^-^ experiments, triplicate glass serum vials (Bellco Glass Inc.) with a total volume of 550 mL each were prepared in an anaerobic glovebox (Coy Laboratories) with CO_2_/H_2_/N_2_ atmosphere (10%/10%/80%). Triplicate bottles were filled with 500 mL of enriched anaerobic microbial culture, either B12^+^ or B12^-^ , in a defined mineral medium as described above. A sterile control containing 500 mL of autoclaved culture (B12^+^ or B12^-^, “killed control” or KC) was prepared to ensure that the isotopic compositions were not affected by experimental design or sampling procedure. Each bottle was amended with a solution of HPLC grade ethanol and lactate (0.3 mL of a solution with 200 mM ethanol and 200 mM lactate) and 41 µL of CF to produce 1 mM aqueous concentration. The CF was isotopically characterized using off-line preparation and dual inlet measurement (δ^13^C= -49.8 ± 0.1‰ expressed with respect to Vienna Pee-Dee-Belemnite, V-PDB^5^). The δ^37^Cl had not been characterized for this CF standard. Bottles were capped with blue butyl stoppers (Bellco Glass Inc.) that were pre-treated by boiling in 0.1M solution of NaOH for 1 hour and rinsed with deionized water.^6^ Triplicate bottles and the controls were stored on their side in the anaerobic chamber for five hours to achieve equilibrium between the aqueous and gaseous phases before analysis, as determined by laboratory protocol tests.

## Detailed Analytical Methods

CF and DCM concentrations were quantified using a Varian 3400 gas chromatograph (GC) equipped with a flame ionization detector (FID) and a RT-Q Bond column (50 m x 0.53 mm ID, 20 μm film thickness). Helium was used as a carrier gas with a column flow of 2 mL/minute. Headspace samples were withdrawn (0.3 mL) using a VICI Pressure-Lok gas tight syringe (Supelco). For each 0.3-mL sample, 0.3 mL of glovebox atmosphere was injected into the vial to maintain constant headspace pressure. The splitless injector temperature was set at 200°C and the detector was set at 210°C. After injecting a sample, the oven temperature was held steady at 100°C for 2 minutes, then ramped up to 175°C at a rate of 25°C/minute and held isothermally for 4 minutes. The oven was then ramped up to 210°C at a rate of 25°C/minute and held isothermally for one minute. Three-point external calibration curves were prepared daily using headspace concentration standards. Relative standard deviations (1σ) for samples and standards using this method were ± 5% based on standard error of the GC measurements.

At each time point of the experiment, 4 mL aqueous samples were taken from each replicate and control bottle for isotope analysis using a glass syringe (Hamilton) and replaced with fresh media (containing vitamin B_12_ in the B12^+^ experiments, and without vitamin B_12_ in the B12^-^ experiments). These samples were added to 5-mL glass vials containing 1 mL of 1 M sulfuric acid to a total volume of 5 mL and capped with a PTFE-lined cap. Samples were then stored at 4ºC upside down during storage and shipment. Prior to analysis, 1-3 mL was taken from the sample vials and added to a 5-mL crimp camp vial, shaken for several minutes, and incubated overnight. Samples were analyzed from the headspace of crimp vials using the method of Heckel et al.^7^

Carbon isotope analyses for the B12^-^ experiments were performed on a Finnigan MAT 252 IRMS interfaced with a Hewlett-Packard 6890 GC via a combustion interface at Toronto (ConFlo I, Isomass). A VOCOL column (Supelco, 30 m x 0.25 mm, 1.8 μm film) was used for separation with a column flow of 2 mL/minute using helium as carrier gas. The injector temperature was set at 200ºC with a split ratio of 1:3. After sample injection, the oven temperature was held steady at 65°C for 1 minute, then ramped up to 175°C at a rate of 25°C/minute and held isothermally for 4 minutes. The oven was ramped up to 160°C at a rate of 5°C/minute, and then ramped to 210°C at a rate of 15°C/minute and held isothermally for one minute. Isotopically characterized in-house working standards of CO_2_ (δ^13^C= -22.3‰, characterized via dual-inlet measurement), CH_4_ (δ^13^C= -39.8‰, characterized via offline preparation and dual-inlet measurement), and a standard mixture of CF (same as described for microcosm amendment) and DCM (δ^13^C= -39.6 ± 0.3‰, characterized via GC-IRMS injections over a range of operating parameters, n =91) were injected daily to ensure measurement accuracy and instrument stability. For both experiments, samples were introduced using headspace analysis^8^ maintaining constant pressure as described above with volumes withdrawn at each time point ranging between 0.3-1 mL. The results of the B12^+^ experiment were completely consistent with previous results on two different IRMS systems^7,9^ (including the instrument used for B12^-^ experiments), as discussed in the results and discussion of the main text. Furthermore, the δ^13^C of the controls, each prepared with the same CF, are consistent between the two instruments thereby confirming that, as expected, no variation in carbon isotope results is due to different labs/instruments. Total uncertainty of δ^13^C measurements incorporating both accuracy and reproducibility is ± 0.5‰.^5,10^

Chlorine isotope measurements were performed on a Neptune MC-ICPMS (Thermo Fisher Scientific, Germany) operated in low resolution mode (m/Δm ≈ 400) with conditions as described in detail by previous papers.^11,12^ A Thermo Scientific Trace 1310 gas chromatographic system was used for separation. Samples were injected using a gastight syringe (Supelco) into a split/splitless injector kept at 250°C with a split ratio of 1:10 or 1:5 and a helium carrier gas flow of 2 mL/minute. A ZB-1 column (Phenomenex, 60 m x 0.32 mm x 1 μm film) was used that was held isothermally at 80°C. Once separated, the compounds entered the plasma via a Thermo Elemental Transferline AE2080 (Aquitaine Electronique, France) heated to 250°C to avoid condensation. Linear regression of the intensity recorded for mass 37 vs. mass 35 at each time point was used to determine the isotopic ratio, where the slope of the best fit line is equal to ^37^Cl/^35^Cl.^13,14^

Raw δ^37^Cl values of the samples were obtained by referencing all compounds versus a methyl chloride working standard which was injected before and after each sequence of samples as has been previously described and authenticated by peer-reviewed method papers^15–17^. In a second step, raw δ^37^Cl values were converted to the international reference scale for chlorine (SMOC) by applying two-point calibration.^15–17^ Three offline characterized in-house standards (measured using DI-IRMS) were used for this procedure: methyl chloride (MC, δ^37^Cl = +6.02‰) and two different trichloroethenes (TCE2, δ^37^Cl = −1.19‰) and TCE6, δ^37^Cl = +2.17 ‰).^18^ Before the measurement of samples started, a sequence of the in-house isotopic standards were injected daily to determine the scale difference compared to DI-IRMS characterized values, used to normalize on the SMOC scale (Eq. S1):

δ^37^Cl_SMOC_= a* δ^37^Cl_RAW_ + b Eq. S1

The slope *a* (scale expansion factor) and intercept *b* (additive correction factor) represent the linear regression of the measured δ^37^Cl_RAW_ of MC and TCE2 (both served as isotopic anchors) plotted versus the offline characterized δ^37^Cl_SMOC_ (“true values”) of these compounds as demonstrated in detail in previous work.^15–17^ For this study, a third standard (TCE6) was used to evaluate the accuracy of this correction procedure, where the two-point calibrated δ^37^Cl_SMOC_ of TCE6 had to agree with the offline value of +2.17 within 0.2‰. The maximum precision (1σ) observed for sample and control measurements was 0.24. As for carbon isotope analyses, since all isotope labs worldwide are calibrated to the same sets of internationally accepted IAEA primary standard materials (V-SMOC in the case of Cl), the results from different labs and different instruments with the same labs should agree (see also discussion in S.I. section 3).

Calculations.

Isotopic enrichment factors were calculated using the Rayleigh model (Eq. 2 in main text). Fraction remaining values were corrected for mass removal.^19^ AKIE values were calculated using Eq. S2:

$\text{AKIE=1/(1+}\text{n}\text{/}\text{x}\text{* }{\text{z}\text{*(}\text{ε}}_{\text{bulk}}\text{/1000)) }$ Eq. S2

where *n* is the total number of atoms in the molecule, *x* is the number of atoms in the reactive position, and *z* is the number of atoms in equivalent reactive positions. For carbon, *n = x = z =1* and for chlorine *n = x = z = 3* for C-Cl bond cleavage in chloroform.

| **Table S-1.** Table showing consistency of ε_C_ values for ACT-3 biotransformation of CF. For discussion of differences in ε_Cl_ values, see Phillips et al.^20^ or S.I. Section 4. | | | |
| --- | --- | --- | --- |
| Study | ε_C_ (‰) | ε_Cl_ (‰) | Λ_C/Cl_ |
| Phillips et al., 2022^20^ | -26.8 ± 3.2 | -6.86 ± 0.35 | 3.39 ± 0.15 |
| Heckel et al., 2019^7^ | -27.91 ± 1.66 | -4.20 ± 0.26 | 6.64 ± 0.14 |
| Chan et al., 2015^9^ | -27.5 ± 0.9 | NA | NA |

## Chlorine isotope results

Since all isotope labs worldwide are calibrated to the same sets of internationally accepted IAEA primary standard materials (V-SMOC in the case of Cl), the results from different labs and different instruments should nonetheless agree within total uncertainty. It is worth noting that for the samples, the chlorine isotope effects determined for B12^+^ experiments (ε_Cl_ = -6.86 ± 0.77‰) are somewhat different (outside uncertainty) compared to the results of Heckel et al.^7^ (ε_Cl_ = -4.20 ± 0.26‰) using the same culture and conditions. These two studies were carried out in two different labs, using different instruments, a GC-IRMS in Heckel et al.^7^ (Munich) and a GC-MC-ICPMS in Phillips et al.^20^ (Leipzig), for chlorine isotope analysis. The use of different mass spectrometers should not affect results and indeed agreement between these two instruments had been previously confirmed by cross-calibration, using two-point calibration with the same international referencing standards; ISL-354 (NaCl, δ^37^Cl = 0.05‰) and USGS38 (KClO_4_, δ^37^Cl = -87.90‰), calibrated on the SMOC scale.^18^ This cross-calibration confirmed consistent measured δ^37^Cl for methyl chloride when two-point calibration using anchors derived from the same international referencing standards is applied.^18^ However, the in-house working standards used for two-point calibration in this work (as described in ^18^) were different from those used by Heckel et al.^21^ Recent work is emphasizing the need for a larger availability of universal standards for δ^37^Cl, so eventually all labs can use not only the same primary IAEA standard (V-SMOC) but the same working standards for daily two-point calibrations.^18^ Further interlaboratory comparison is necessary to identify the cause of variation between these two studies, however this is beyond the scope of this work. Fortunately, for all the work presented here, all data were run on the same instrument and with the same two-point calibration standards, and as such complete inter-comparability of results is ensured. This issue is described simply to allow comparison to previous work (specifically by Heckel et al.^7^).

## Experimental Results for Sterile Controls

Carbon Isotope Measurements

| 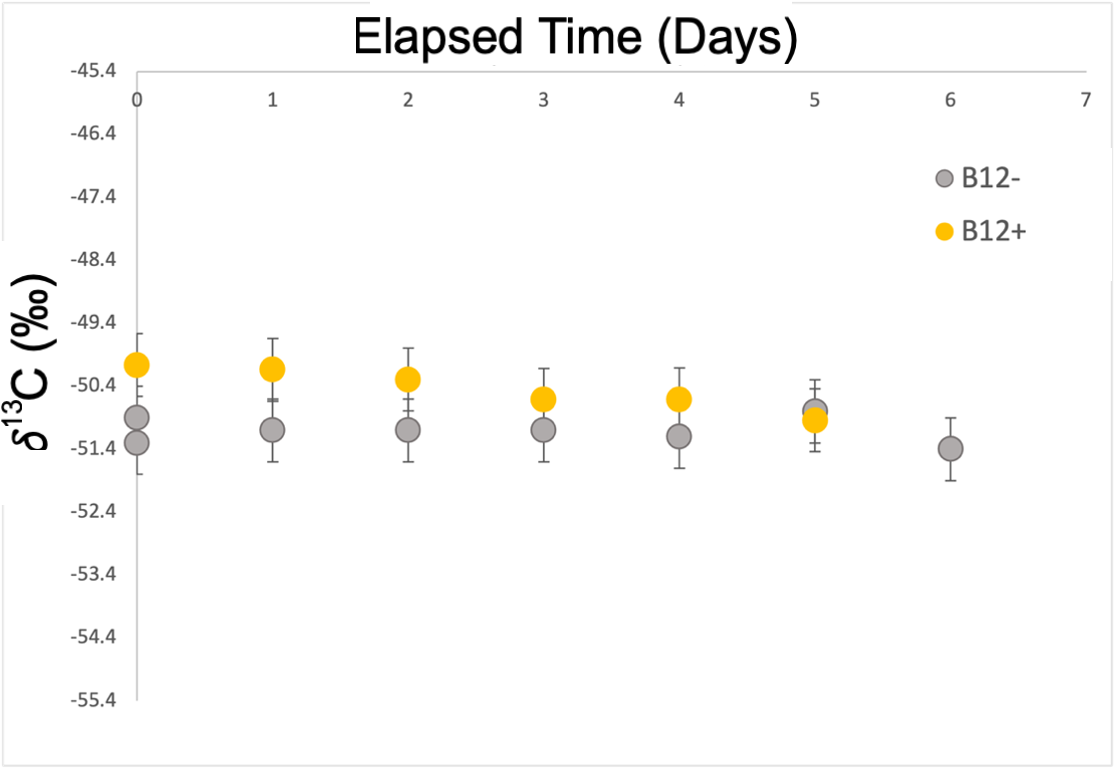 |
| --- |
| **Figure S-1.** Plot of δ^13^C (‰) vs. elapsed time in days for the sterile controls for the B12- (grey) and B12+ (yellow) anaerobic biotransformation experiment. Error bars on the y-axis represent total analytical uncertainty for carbon CSIA of 0.5‰ after Sherwood Lollar et al.^22^. No change outside of uncertainty is observed in any of the control vials. |

| Chlorine Isotope Measurements  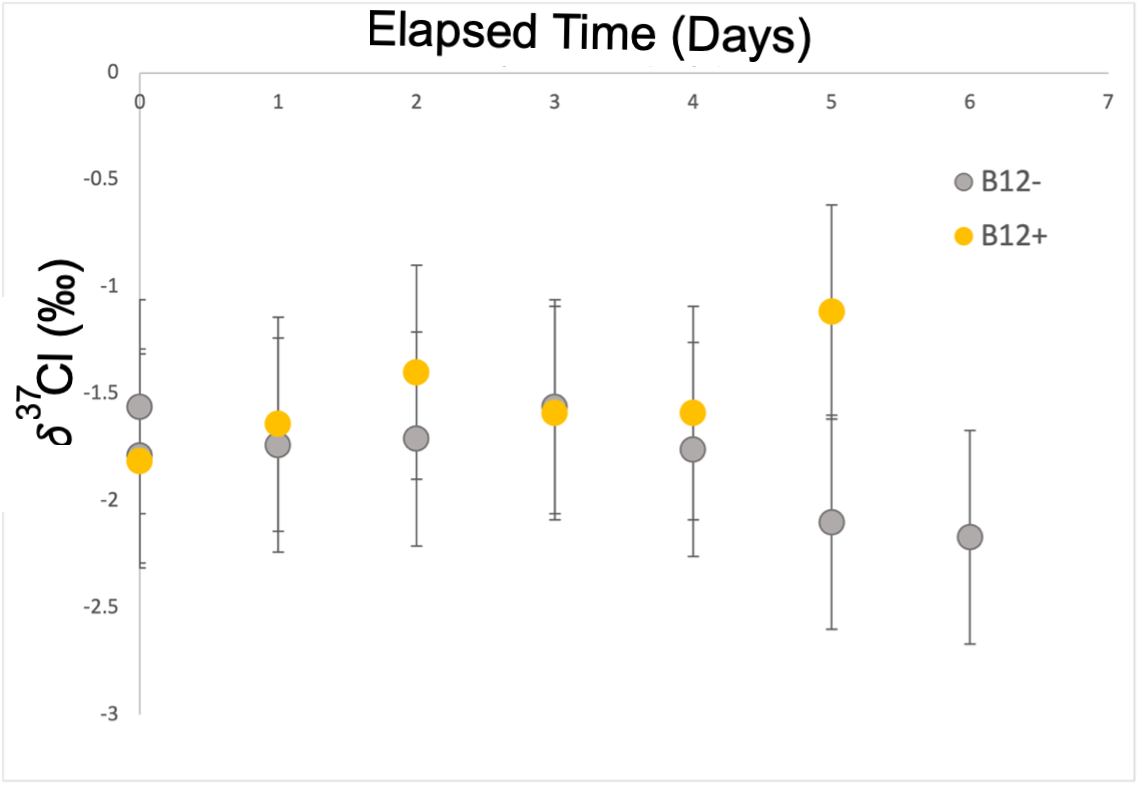 |
| --- |
| **Figure S-2.** Plot of δ^37^Cl (‰) vs. elapsed time in days for the sterile controls for the B12- (grey) and B12+ (yellow) anaerobic biotransformation experiment. Error bars on the y-axis represent total analytical uncertainty for chlorine CSIA of 0.5‰ based on 2σ precision of standard and control measurements.   \|  \| \| --- \| \|  \| |

## Experimental Data: Mass vs. Time

| 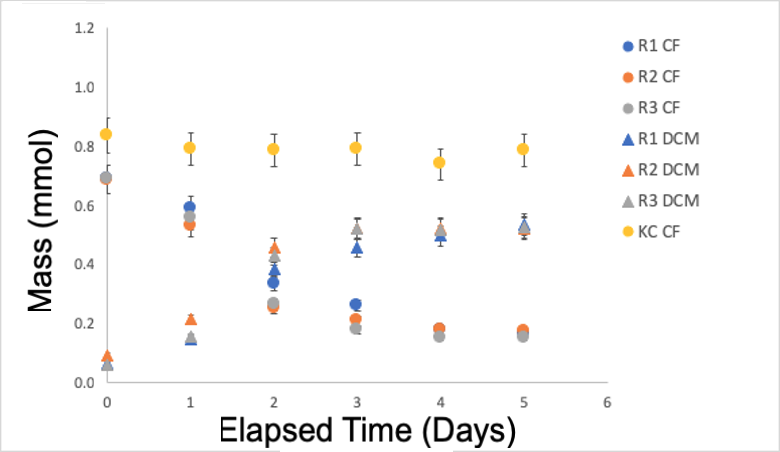 |
| --- |
| **Figure S‑3.** Plot of mass (mmol) of CF and DCM vs. elapsed time (in days) for all replicates in the B12^+^ anaerobic biotransformation experiment. Error bars on the y-axis represent propagated error of ±7% (using standard error propagation). The mass balance (sum of CF and DCM mass in mmol) was consistent over the course of the experiment. |

| \| 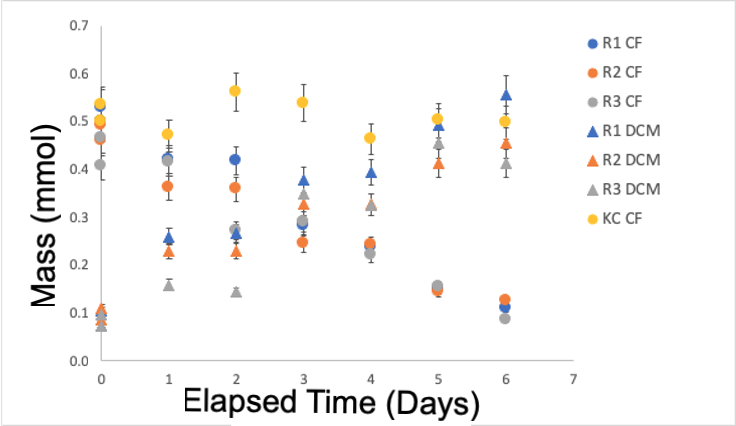 \| \| --- \| \| **Figure S‑4.** Plot of mass (mmol) of CF and DCM vs. elapsed time (in days) for all replicates in the B12^-^ anaerobic biotransformation experiment. Error bars on the y-axis represent propagated error of ±7% (using standard error propagation). The mass balance (sum of CF and DCM mass in mmol) was consistent over the course of the experiment. \| |
| --- | --- | --- |

## Community Composition in B12^+^ vs. B12^-^

**Figure S-5.** Community composition of ACT-3 CF/ B12^+^ and ACT-3 CF/ B12^-^. Bacteria (A) and Archaea (B) are visualized separately to account for kingdom-specific sequencing biases. Each colour represents one amplicon sequence variant. All ASVs are summarized in Table S-4.

1. Metagenomic sequencing, assembly, and cobalamin synthesis search

**Table S-2.** Steps of anaerobic cobalamin synthesis pathway, and their respective Hidden Markov Models (HMMs) searched for and detected in the ACT-3 CF metagenome.

| **Step Code** | **Step Name** | **HMM*** | **Hits** |
| --- | --- | --- | --- |
| CbiA | cobyrinic acid a,c-diamide synthase | [TIGR00379](http://tigrfams.jcvi.org/cgi-bin/HmmReportPage.cgi?acc=TIGR00379) | 12 |
| CbiC | precorrin-8X methylmutase | [PF02570](http://pfam.sanger.ac.uk/family/PF02570) | 17 |
| CbiD | cobalamin biosynthesis protein CbiD | [TIGR00312](http://tigrfams.jcvi.org/cgi-bin/HmmReportPage.cgi?acc=TIGR00312) | 9 |
| CbiD | CbiD (decarboxylating), CbiT subunit | [PF01888](http://pfam.sanger.ac.uk/family/PF01888) | 27 |
| CbiE | precorrin-6y C5,15-methyltransferase | [TIGR02467](http://tigrfams.jcvi.org/cgi-bin/HmmReportPage.cgi?acc=TIGR02467) | 12 |
| CbiF | precorrin-4 C11-methyltransferase | [TIGR01465](http://tigrfams.jcvi.org/cgi-bin/HmmReportPage.cgi?acc=TIGR01465) | 9 |
| CbiG | cobalamin biosynthesis protein, C-terminal | [PF01890](http://pfam.sanger.ac.uk/family/PF01890) | 15 |
| CbiH | precorrin-3B C17-methyltransferase | [TIGR01466](http://tigrfams.jcvi.org/cgi-bin/HmmReportPage.cgi?acc=TIGR01466) | 11 |
| CbiJ | precorrin-6x reductase CbiJ/CobK | [PF02571](http://pfam.sanger.ac.uk/family/PF02571) | 22 |
| CbiJ | precorrin-6x reductase | [TIGR00715](http://tigrfams.jcvi.org/cgi-bin/HmmReportPage.cgi?acc=TIGR00715) | 8 |
| CbiK/X | sirohydrochlorin cobaltochelatase | [PF01903](http://pfam.sanger.ac.uk/family/PF01903) | 20 |
| CbiK/X | cobalt chelatase CbiK | [PF06180](http://pfam.sanger.ac.uk/family/PF06180) | 23 |
| CbiL | precorrin-2 C | [TIGR01467](http://tigrfams.jcvi.org/cgi-bin/HmmReportPage.cgi?acc=TIGR01467) | 8 |
| CbiT | precorrin-6Y C5,15-methyltransferase (20)-methyltransferase | [TIGR02469](http://tigrfams.jcvi.org/cgi-bin/HmmReportPage.cgi?acc=TIGR02469) | 10 |
| CobA | uroporphyrinogen-III C-methyltransferase (decarboxylating), CbiT subunit | [TIGR01469](http://tigrfams.jcvi.org/cgi-bin/HmmReportPage.cgi?acc=TIGR01469) | 11 |
| CobA | cobyrinate adenosyltransferases (BtuR, PduO, EutT) | [PF01923](http://tigrfams.jcvi.org/cgi-bin/HmmReportPage.cgi?acc=TIGR00708) | 26 |
| CobU | bifunctional cobinamide kinase, cobinamide phosphate guanylyltransferase (CobP) | [PF02283](http://tigrfams.jcvi.org/cgi-bin/HmmReportPage.cgi?acc=TIGR00317) | 25 |
| CobS | cobalamine 5-phosphate synthase (alpha ribazole transferase) (CobV) | [PF02654](http://pfam.sanger.ac.uk/family/PF03186) | 31 |
| CobD | adenosylcobinamide synthase (CobD) | [PF03186](http://tigrfams.jcvi.org/cgi-bin/HmmReportPage.cgi?acc=TIGR03161) | 26 |
| CobQ | cobyric acid synthase (CobQ/CbiP) | [TIGR00313](http://tigrfams.jcvi.org/cgi-bin/HmmReportPage.cgi?acc=TIGR03162) | 14 |
| CobS | cobalamine 5-phosphate synthase (alpha ribazole transferase) (CobV) | [TIGR00317](http://tigrfams.jcvi.org/cgi-bin/HmmReportPage.cgi?acc=TIGR00313) | 8 |
| AcbPsyn | adenosylcobinamide-phosphate synthase | TIGR00380 | 13 |
| CobA | cobyrinate adenosyltransferases (BtuR, PduO, EutT) | [TIGR00636](http://tigrfams.jcvi.org/cgi-bin/HmmReportPage.cgi?acc=TIGR00380) | 13 |
| CobA | cobyrinate adenosyltransferases (BtuR, PduO, EutT) | TIGR00708 | 4 |
| ThrP_dc | threonine-phosphate decarboxylase | [TIGR01140](http://tigrfams.jcvi.org/cgi-bin/HmmReportPage.cgi?acc=TIGR03160) | 5 |
| CobT | nicotinate nucleotide-DBI P-ribosyltransferase | [TIGR03160](http://pfam.sanger.ac.uk/family/PF02654) | 13 |
| CobC/Z | alpha-ribazole-5`-phosphate phosphatase | [TIGR03161](http://tigrfams.jcvi.org/cgi-bin/HmmReportPage.cgi?acc=TIGR00636) | 0 |
| CobC/Z | alpha-ribazole-5`-phosphate phosphatase | [TIGR03162](http://pfam.sanger.ac.uk/family/PF01923) | 2 |

* for more information, see [here](http://genome-properties.jcvi.org/cgi-bin/GenomePropDefinition.cgi?prop_acc=GenProp0274)

See Tables S-3 and S-4 in attached file.

References

(1) Grostern, A.; Edwards, E. A. A 1,1,1-Trichloroethane-Degrading Anaerobic Mixed Microbial Culture Enhances Biotransformation of Mixtures of Chlorinated Ethenes and Ethanes. *Appl Environ Microbiol* **2006**, *72* (12), 7849–7856. https://doi.org/10.1128/AEM.01269-06.

(2) Duhamel, M.; Grostern, A.; Dworatzek, S.; Edwards, E. A. Chloroform Respiration to Dichloromethane by a Dehalobacter Population. *Environ Microbiol* **2010**, *12* (4), 1053–1060. https://doi.org/10.1111/j.1462-2920.2009.02150.x.

(3) Tang, S.; Gong, Y.; Edwards, E. A. Semi-Automatic In Silico Gap Closure Enabled De Novo Assembly of Two *Dehalobacter* Genomes from Metagenomic Data. *PLoS One* **2012**, *7* (12). https://doi.org/10.1371/journal.pone.0052038.

(4) Wang, P. H.; Tang, S.; Nemr, K.; Flick, R.; Yan, J.; Mahadevan, R.; F Yakunin, A.; Löffler, F. E.; Edwards, E. A. Refined Experimental Annotation Reveals Conserved Corrinoid Autotrophy in Chloroform-Respiring *Dehalobacter* Isolates. *ISME Journal* **2017**, *11* (3), 626–640. https://doi.org/10.1038/ismej.2016.158.

(5) Hunkeler, D.; Meckenstock, R. U.; Sherwood Lollar, B.; Schmidt, T. C.; Wilson, J. T. A Guide for Assessing Biodegradation and Source Identification of Organic Ground Water Contaminants Using Compound Specific Isotope Analysis (CSIA). *USEPA Publication* **2008**, *EPA 600/R-* (December), 1–82. https://doi.org/EPA/600/R-08/148.

(6) Ward, J. A.; Slater, G. F.; Moser, D. P.; Lin, L. H.; Lacrampe-Couloume, G.; Bonin, A. S.; Davidson, M.; Hall, J. A.; Mislowack, B.; Bellamy, R. E. S.; Onstott, T. C.; Sherwood Lollar, B. Microbial Hydrocarbon Gases in the Witwatersrand Basin, South Africa: Implications for the Deep Biosphere. *Geochim Cosmochim Acta* **2004**, *68* (15), 3239–3250. https://doi.org/10.1016/j.gca.2004.02.020.

(7) Heckel, B.; Phillips, E.; Edwards, E.; Sherwood Lollar, B.; Elsner, M.; Manefield, M. J.; Lee, M. Reductive Dehalogenation of Trichloromethane by Two Different *Dehalobacter Restrictus* Strains Reveal Opposing Dual Element Isotope Effects. *Environ Sci Technol* **2019**, *53* (5), 2332–2343. https://doi.org/10.1021/acs.est.8b03717.

(8) Slater, G. F.; Dempster, H. S.; Sherwood Lollar, B.; Ahad, J. Headspace Analysis: A New Application for Isotopic Characterization of Dissolved Organic Contaminants. *Environ. Sci. Technol.* **1999**, *33* (1), 190–194. https://doi.org/10.1021/es9803254.

(9) Chan, C. C. H.; Mundle, S. O. C.; Eckert, T.; Liang, X.; Tang, S.; Lacrampe-Couloume, G.; Edwards, E. A.; Sherwood Lollar, B. Large Carbon Isotope Fractionation during Biodegradation of Chloroform by *Dehalobacter* Cultures. *Environ Sci Technol* **2012**, *46* (18), 10154–10160. https://doi.org/10.1021/es3010317.

(10) Sherwood Lollar, B.; Hirschorn, S. K.; Chartrand, M. M. G.; Lacrampe-Couloume, G. An Approach for Assessing Total Instrumental Uncertainty in Compound-Specific Carbon Isotope Analysis: Implications for Environmental Remediation Studies. *Anal Chem* **2007**, *79* (9), 3469–3475. https://doi.org/10.1021/ac062299v.

(11) Horst, A.; Renpenning, J.; Richnow, H.; Gehre, M. Compound Specific Stable Chlorine Isotopic Analysis of Volatile Aliphatic Compounds Using Gas Chromatography Hyphenated with Multiple Collector Inductively Coupled Plasma Mass Spectrometry. *Anal Chem* **2017**, *89*, 9131–9138. https://doi.org/10.1021/acs.analchem.7b01875.

(12) Renpenning, J.; Horst, A.; Schmidt, M.; Gehre, M. Online Isotope Analysis of ^37^Cl/^35^Cl Universally Applied for Semi-Volatile Organic Compounds Using GC-MC-ICPMS. *J Anal At Spectrom* **2018**, *33* (2), 314–321. https://doi.org/10.1039/c7ja00404d.

(13) Fietzke, J.; Frische, M.; Hansteen, T. H.; Eisenhauer, A. A Simplified Procedure for the Determination of Stable Chlorine Isotope Ratios (δ^37^Cl) Using LA-MC-ICP-MS. *J Anal At Spectrom* **2008**, *23* (5), 769–772. https://doi.org/10.1039/b718597a.

(14) Epov, V. N.; Rodriguez-Gonzalez, P.; Sonke, J. E.; Tessier, E.; Amouroux, D.; Bourgoin, L. M.; Donard, O. F. X. Simultaneous Determination of Species-Specific Isotopic Composition of Hg by Gas Chromatography Coupled to Multicollector ICPMS. *Anal Chem* **2008**, *80* (10), 3530–3538. https://doi.org/10.1021/ac800384b.

(15) Renpenning, J.; L. Hitzfeld, K.; Gilevska, T.; Nijenhuis, I.; Gehre, M.; Richnow, H.-H. Development and Validation of an Universal Interface for Compound-Specific Stable Isotope Analysis of Chlorine (^37^Cl/^35^Cl)) by GC-High-Temperature Conversion (HTC)-MS/IRMS. *Anal Chem* **2015**, *87* (5), 2832–2839. https://doi.org/10.1021/ac504232u.

(16) Coplen, T. B. Normalization of Oxygen and Hydrogen Isotope Data. *Chemical Geology: Isotope Geoscience Section* **1988**, *72* (4), 293–297. https://doi.org/10.1016/0168-9622(88)90042-5.

(17) Paul, D.; Skzypek, G.; Forizas, I. Normalization of Measured Stable Isotopic Compositions to Isotope Reference Scales - a Review. *Rapid Communications in Mass Spectrometry* **2007**, *21*, 3006–3014. https://doi.org/10.1002/rcm.

(18) Lihl, C.; Renpenning, J.; Kümmel, S.; Gelman, F.; K. V. Schürner, H.; Daubmeier, M.; Heckel, B.; Melsbach, A.; Bernstein, A.; Shouakar-Stash, O.; Gehre, M.; Elsner, M. Toward Improved Accuracy in Chlorine Isotope Analysis: Synthesis Routes for In-House Standards and Characterization via Complementary Mass Spectrometry Methods. *Anal Chem* **2019**, *91* (19), 12290–12297. https://doi.org/10.1021/acs.analchem.9b02463.

(19) Buchner, D.; Jin, B.; Ebert, K.; Rolle, M.; Elsner, M.; Haderlein, S. B. Experimental Determination of Isotope Enrichment Factors - Bias from Mass Removal by Repetitive Sampling. *Environ Sci Technol* **2017**, *51* (3), 1527–1536. https://doi.org/10.1021/acs.est.6b03689.

(20) Phillips, E.; Bulka, O.; Picott, K.; Kümmel, S.; Edwards, E. A.; Nijenhuis, I.; Gehre, M.; Dworatzek, S.; Webb, J.; Sherwood Lollar, B. Investigation of Active Site Amino Acid Influence on Carbon and Chlorine Isotope Fractionation during Reductive Dechlorination. *FEMS Microbiol Ecol* **2022**, *98* (8). https://doi.org/10.1093/femsec/fiac072.

(21) Heckel, B.; Phillips, E.; Edwards, E.; Sherwood Lollar, B.; Elsner, M.; Manefield, M.; Lee, M. Reductive Dehalogenation of Trichloromethane by Two Different *Dehalobacter Restrictus* Strains Reveal Opposing Dual Element Isotope Effects. *Environ Sci Technol* *53* (5), 2332–2343. https://doi.org/10.1021/acs.est.8b03717.

(22) Sherwood Lollar, B.; Hirschorn, S. K.; Chartrand, M. M. G.; Lacrampe-Couloume, G. An Approach for Assessing Total Instrumental Uncertainty in Compound-Specific Carbon Isotope Analysis: Implications for Environmental Remediation Studies. *Analytical Chemistry* **2007**, *79* (9), 3469–3475. https://doi.org/10.1021/ac062299v.
